# The relation between dietary phytochemical index and metabolic health status in overweight and obese adolescents

**DOI:** 10.1038/s41598-023-39314-z

**Published:** 2023-07-25

**Authors:** Shahnaz Amani Tirani, Keyhan Lotfi, Saeideh Mirzaei, Ali Asadi, Masoumeh Akhlaghi, Parvane Saneei

**Affiliations:** 1 Department of Community Nutrition, School of Nutrition and Food Science, Nutrition and Food Security Research Center, Students’ Research Committee, Isfahan University of Medical Sciences, PO Box 81745-151, Isfahan, Iran; 2 Department of Community Nutrition, School of Nutritional Sciences and Dietetics, Tehran University of Medical Sciences, Tehran, Iran; 3 Department of Community Nutrition, School of Nutrition and Food Science, Shiraz University of Medical Sciences, Shiraz, Iran; 4 Department of Exercise Physiology, School of Physical Education and Sport Sciences, University of Tehran, Tehran, Iran; 5 Department of Community Nutrition, School of Nutrition and Food Science, Nutrition and Food Security Research Center, Isfahan University of Medical Sciences, PO Box 81745-151, Isfahan, Iran; 6 Students’ Scientific Research Center, Tehran University of Medical Sciences, Tehran, Iran

**Keywords:** Endocrinology, Health care, Medical research

## Abstract

Previous studies have rarely investigated dietary phytochemicals consumption in relation to metabolic health of adolescents. The current study was performed to investigate dietary phytochemical index (DPI) in relation to metabolic health status in overweight and obese adolescents. This cross-sectional study was conducted among 203 adolescents with overweight or obesity. Dietary intakes of participants were obtained through a validated 147-item food frequency questionnaire. DPI was calculated [(dietary energy derived from phytochemical-rich foods (kcal)/total daily energy intake (kcal)) ⨯100]. Glycemic and lipid profiles, blood pressure, and anthropometric indices were also measured. A metabolically unhealthy overweight/obesity (MUO) profile was determined based on the International Diabetes Federation (IDF) and IDF/Homeostasis Model Assessment Insulin Resistance (HOMA-IR) definitions. Study subjects had a mean age of 13.98 years and 50.2% of them were girls. According to IDF and IDF/HOMA-IR criteria, 38.9% (37 boys, and 42 girls) and 33% (35 boys, and 32 girls) of the study participants were respectively MUO. According to IDF and IDF/HOMA-IR definitions, adolescents in the third DPI tertile had respectively 61% (maximally-adjusted OR = 0.39, 95%CI 0.16–0.91) and 67% (maximally-adjusted OR = 0.33, 95%CI 0.13–0.83) lower odds of being MUO, compared to the first tertile. Stratified analysis by sex indicated that DPI was inversely related to MUO phenotype based on IDF criteria in girls (maximally-adjusted OR = 0.25, 95%CI 0.06–0.98), but not in boys. The current study found that adolescents with a higher dietary intake of phytochemicals have lower odds of being MUO, particularly among girls. However, further large-scale prospective cohort studies are required to confirm this finding.

## Introduction

Public health concern has been raised toward the increasing prevalence of obesity among children and adolescents in the recent decades^1^. Adolescent obesity increases the risk of premature death and cardiovascular risk factors, including hyperlipidemia, hypertension, diabetes mellitus, and non-alcoholic fatty liver^2,3^. Some evidences have also suggested that increased body mass index (BMI) in adolescence is related to an elevated risk of various types of cancers in adulthood^4–6^. Furthermore, obese adolescents suffer from mental disorders such as poor self-esteem, anxiety, and depression^7–9^. Therefore, the effective prevention and management of obesity in childhood should be considered as a public health priority.

Among obese individuals, those without cardiometabolic risk factors are considered as "metabolically healthy obese" (MHO)^10^. There is a substantial variation in reported MHO prevalence, due to differences in definitions used to define this condition^11^. Despite being obese, MHO adolescents have a normal metabolic status, including normal insulin sensitivity, blood lipids, blood pressure (BP), and blood glucose^12^. It has been demonstrated that if MHO adolescents could maintain their favorable cardiometabolic profile into adulthood, they would have a lower risk of cardiovascular diseases^13^. Thus, exploring factors which could be beneficial in maintaining MHO phenotype from adolescence to adulthood is of particular importance.

Dietary factors are among modifiable determinants of the metabolic status in overweight/obese adolescents^14,15^. Previous investigations have identified an inverse association between higher intake of several food groups including nuts, vegetables, fruits, and whole grains with cardiometabolic risk in adolescents^16–18^. These plant-based foods possibly exert their beneficial role through vitamins, minerals, phytochemicals, and dietary fiber. Phytochemicals are non-nutritive bioactive compounds with a striking diversity in function and structure. The most important dietary phytochemicals are polyphenols, carotenoids, isoprenoids, phytosterols, saponins, organosulfur compounds, and dietary fibers^19^. Phytochemicals are found in plant-based foods such as vegetables, fruits, nuts, whole grains, and herbs which have received considerable attention, due to their health-promoting influences^20,21^.

Dietary phytochemical index (DPI) is an alternative feasible approach presented by McCarty to estimate dietary phytochemical content^22^. DPI is defined as the percent of dietary calorie obtained from foods with a high phytochemical content^22^. Earlier observational studies have examined the relation between some phytochemicals such as polyphenols and flavonoids with cardiometabolic risk factors and metabolic syndrome (MetS) in adolescent subjects^23,24^. However, no prior study has investigated the whole DPI in relation metabolic health status of adolescents. We hypothesized that higher DPI might be in relation to a better metabolic health status of adolescents. Thus, we conducted the current study to investigate the relation between DPI and metabolic health status in Iranian overweight and obese adolescents.

## Methods

### Study design and participants

A representative sample of Iranian adolescents (12–18 years old) participated in this cross-sectional study. Participants were randomly selected by a stratified, multistage cluster sampling method from several middle and high schools of various education districts of Isfahan, Iran. According to the age-sex specific percentile curves of BMI^25^, overweight/obese students were screened and invited to the study. The required sample size was estimated using earlier investigations in which a prevalence of 60% for MUO had been reported among Iranian overweight/obese adolescents^26^. The minimum sample size was estimated to be 188, considering a power of 80%, type I error of 0.05, desired confidence interval (CI) of 0.95, and precision (d) of 7%. Subjects with a specific genetic or endocrine condition including hypothyroidism, type 1 diabetes mellitus, and Cushing’s syndrome were excluded. Also, those who adhered to a weight-loss diet, and took nutritional supplements or medications that might influence lipid profile, body weight, blood pressure or blood glucose were excluded. Finally, 203 overweight/obese adolescents including 102 girls and 101 boys were eligible to include in the analysis. The study was performed in accordance with the Declaration of Helsinki and approved by the ethics committee of the Isfahan University of Medical Sciences (no.2101219). All participants and their parents signed informed consent forms.

### Dietary intakes

Dietary intakes of individuals during the preceding year were obtained by a validated 147-item food frequency questionnaire (FFQ)^27,28^. The reasonable validity and reliability of the applied FFQ for assessing the food intake of Iranian adolescents have been documented by earlier investigations^29,30^. An interviewer asked participants to report their intake of each food group per day, week, or month. Standard portion sizes were used to report the amount of the consumed foods. Then, reported portion sizes were converted to grams/day according to the household measures which were previously defined^31^. Daily intakes of energy and nutrients were computed through Nutritionist IV software.

### Phytochemical index calculation

PDI for each participant was computed according to the following equation suggested by McCarty^22^: DPI = [(dietary energy derived from phytochemical-rich foods (kcal)/ total daily energy intake (kcal)) ⨯100]. Phytochemical-rich foods were considered as: vegetables (starchy vegetables, red, dark green, orange vegetables, and other vegetables), fruits (orange, yellow, and red fruits), natural vegetable and fruit juices as well as tomato sauces, whole grains (traditional Iranian breads (Barbari and Sangak bread), oat, and bulgur), legumes (chickpea, beans, lentil, Vicia faba, soy, mung bean and split bean), nuts (peanut, almond, walnut, hazelnut, and pistachio), seeds, olives, and olive oil. As potato has a limited content of phytochemicals, we did not consider this item for DPI calculation.

### Anthropometric indices and cardio-metabolic risk factors

An expert nutritionist evaluated anthropometric factors according to standard protocols. Body weight was measured with minimal clothes and without shoes to the nearest 0.1 kg by a digital scale (Seca Instruments, Germany). Also, height was measured in a standing status, while having no shoes using a stadiometer. BMI was computed by dividing weight (kg) by squared height (m^2^). Then, we classified participants according to the World Health Organization (WHO) growth curve of age-sex-specific BMI percentiles to normal weight (5th < BMI < 85th percentile), overweight (85th < BMI < 95th percentile), or obese (BMI > 95th percentile)^25^. Only overweight/obese students were invited to this investigation. Waist circumference was also measured two times following a normal expiration and without any pressure on the body surface, midway between the lowest rib and the superior border of the iliac crest. After asking participants to have a rest of 15 min, blood pressure was measured twice on the right arm, with a 5-min interval, by a mercury sphygmomanometer.

Twelve-hour fasting blood samples were collected for biochemical indices evaluation. Fasting blood glucose (FBG) concentration was assessed based on an enzymatic colorimetric method using a commercial kit (Pars Azmoon commercial kits, Tehran, Iran). In addition, serum levels of insulin were examined using ELISA kit (Diagnostic Biochem Canada Inc.). Insulin resistance was assessed according to the homeostasis model assessment insulin resistance (HOMA-IR) using the following formula: HOMA-IR = [(fasting insulin (mU/L) × FBG (mmol/L)]/22.5. We measured serum concentrations of high-density lipoprotein cholesterol (HDL-c) and triglyceride (TG) by commercial kits (Pars Azmoon commercial kits, Tehran, Iran).

### Metabolic health status

Two distinct definitions were applied for classifying individuals to MHO and MUO. In first classification, we used the International Diabetes Federation (IDF) criteria that considered subjects with the presence of ≥ 2 of the following risk factors as MUO: elevated TG (≥ 150 mg/dL), reduced HDL-c (< 40 mg/dL for the age of < 16 y, and < 50 mg/dL in girls/ < 40 mg/dL in boys for the age of ≥ 16 y), elevated FBG (≥ 100 mg/dL) and elevated blood pressure (≥ 130/85 mmHg)^32^. The second classification was similar to the IDF criteria, along with considering the presence of insulin resistance (IR). In this definition, subjects were defined as MUO if they had HOMA-IR ≥ 3.16 and at least two of the aforementioned risk factors of IDF criteria. In opposite, subjects with HOMA-IR lower than 3.16 (regardless of the number of cardiometabolic risk factors) were considered as MHO^33^.

### Other variables

A 9-item validated physical activity questionnaire for adolescents (PAQ-A) was used to examine the physical activity of participants^34,35^. The first eight items evaluate usual activities with a 5-point rating answer, whereas the last item estimates unusual activities of study subjects in the last week. Students were classified as sedentary (or having no regular physical activity) (score < 2), low active (2 ≤ score < 3), moderately active (3 ≤ score < 4), and highly active (score ≥ 4), according to the total summed score of all questions. As we had few sedentary and high active subjects, the first and the last two categories were combined to their adjacent categories to have two physical activity categories (sedentary and low active/ moderately and highly active). A standard questionnaire was also used to gather information regarding age, sex, disease history, and consumed supplements or medications. Data of family size, parental education level, parental job, having cars in the family, having a personal room, having computers/laptops, and taking trips in the last year (which were considered as socioeconomic variables) were collected by a validated questionnaire^36^.

### Statistical analysis

First, subjects were categorized according to tertiles of DPI (T_1_: < 11.50, T_2_: 11.50–21.90, T_3_: > 21.90). For general characteristics of participants, categorical and continuous variables were respectively reported as frequency (percentage) and mean ± SD. The chi-square test and analysis of variance (ANOVA) determined the differences of categorical and continuous variables across tertiles of DPI, respectively. Furthermore, we examined age, sex, and energy-adjusted dietary intakes of subjects across tertiles of DPI by the analysis of covariance (ANCOVA). In the ANCOVA, energy intake and macronutrients (as percentage of total energy intake) were only adjusted for sex and age. Binary logistic regression in crude and multivariable-adjusted models was applied to assess the association between DPI and odds of being MUO by reporting odds ratios (ORs) and the corresponding 95% confidence intervals (95% CIs). In the first adjusted model, age, sex, and energy intake were considered. In the second model, physical activity and socioeconomic variables were adjusted, as well. Finally, in the third model, BMI was additionally adjusted. The first tertile of DPI was considered as the reference category in all models. To specify the trend of odds ratios, tertiles of DPI were considered as an ordinal variable in all logistic regression models. Furthermore, stratified analyses were undertaken according to sex. We conducted data analysis using SPSS version 20 software. P-values < 0.05 were regarded as significant.

## Results

In total, 203 adolescents including 101 boys and 102 girls with a mean age of 13.98 ± 1.61 (SD) years participated in the present study. MUO phenotype was identified in 38.9% (37 boys, and 42 girls) and 33% (35 boys, and 32 girls) subjects according to IDF criteria, and IDF/HOMA-IR definition, respectively.

Table 1 summarizes general characteristics and cardiometabolic factors of adolescents across tertiles of DPI. The mean BMI (P = 0.02), WC (P = 0.01), FBG (P = 0.002), insulin (P = 0.01), and HOMA-IR (P = 0.01) were significantly lower among participants in the highest tertile of DPI in comparison to those in the lowest tertile. Furthermore, adolescents in the third tertile of DPI had a marginally lower mean age (P = 0.06), DBP (P = 0.09), and TG (P = 0.09) than those in the first tertile. However, participants in the highest tertile of DPI had a significantly higher level of HDL-c, compared to those in the lowest tertile (P = 0.02). In addition, significant differences were observed in physical activity (P < 0.001) and socioeconomic status of participants (P = 0.04) across tertiles of DPI.

**Table 1:** General characteristics and cardiometabolic factors of study participants across tertiles of DPI^1^.

DPI tertiles				
	T1 (n = 67) (< 11.50)	T2 (n = 68) (11.50–21.90)	T3 (n = 68) (> 21.90)	P-value^2^
Sex, n (%)				0.42
Boys	30 (44.8)	33 (48.5)	38 (55.9)	
Girls	37 (55.2)	35 (51.5)	30 (44.1)	
Age (year)	14.31 $\pm$ 1.56	13.97 $\pm$ 1.47	13.66 $\pm$ 1.73	0.06
Weight (kg)	76.31 $\pm$ 11.78	74.88 $\pm$ 12.08	69.29 $\pm$ 9.77	0.001
Height (cm)	165.08 $\pm$ 8.61	164.21 $\pm$ 7.74	161.62 $\pm$ 7.14	0.03
BMI (kg/m^2^)	27.89 $\pm$ 2.89	27.71 $\pm$ 3.70	26.47 $\pm$ 2.92	0.02
Waist circumference (cm)	92.10 $\pm$ 7.70	90.83 $\pm$ 7.55	88.08 $\pm$ 8.12	0.01
Physical activity levels, n (%)				< 0.001
Low	65 (98.5)	57 (83.8)	43 (63.2)	
High	2 (1.5)	11 (16.2)	25 (36.8)	
Socioeconomic status^3^, n (%)				0.04
Low	28 (41.8)	17 (25.0)	14 (20.6)	
Medium	26 (38.8)	34 (50.0)	30 (44.1)	
High	13 (19.4)	17 (25.0)	24 (35.3)	
Systolic blood pressure (mmHg)	114.27 $\pm$ 17.05	112.01 $\pm$ 20.78	111.85 $\pm$ 17.13	0.69
Diastolic blood pressure (mmHg)	75.46 $\pm$ 10.38	73.88 $\pm$ 10.09	71.17 $\pm$ 13.13	0.09
Fasting blood glucose (mg/dL)	100.33 $\pm$ 9.56	98.79 $\pm$ 6.85	95.31 $\pm$ 8.24	0.002
Insulin (μUI/mL)	23.97 $\pm$ 14.62	19.57 $\pm$ 9.33	17.78 $\pm$ 12.84	0.01
HOMA-IR index	5.92 $\pm$ 3.57	4.82 $\pm$ 2.45	4.33 $\pm$ 3.53	0.01
Triglycerides (mg/dL)	134.99 $\pm$ 70.86	121.35 $\pm$ 63.79	109.71 $\pm$ 63.38	0.09
HDL-c (mg/dL)	42.96 $\pm$ 7.91	44.65 $\pm$ 8.03	46.84 $\pm$ 7.45	0.02

1 Values are Mean ± SD; unless indicated. Abbreviations: DPI: dietary phytochemical index; BMI: Body Mass Index; HOMA-IR: Homeostasis Model Assessment Insulin Resistance; HDL-c: high-density lipoprotein cholesterol.

2 Obtained from one-way ANOVA and χ2 test for quantitative and categorical variables, respectively.

3 Socioeconomic status (SES) score was evaluated based on parental education level, parental job, family size, having car in the family, having computer/laptop, having personal room and having travel by using a validated questionnaire.

Dietary intakes of macro-and micro-nutrients of study individuals across tertiles of DPI are presented in Table 2. Adolescents in the third tertile of DPI had a significantly higher intake of protein, vitamin C, riboflavin, vitamin B6, folate, vitamin B12, magnesium, calcium, total fiber, and vitamin K in comparison to those in the first tertile (P < 0.001 for all variables). However, participants in the highest tertile of DPI had a significantly lower intake of polyunsaturated fatty acid (PUFA) (P = 0.02), vitamin A, vitamin E, and niacin (P < 0.001 for all 3 variables).

**Table 2:** Dietary intakes (energy and macro/micro nutrients) of study participants across energy-adjusted tertiles of DPI^1^.

DPI tertiles				
	T1 (n = 67) (< 11.50)	T2 (n = 68) (11.50–21.90)	T3 (n = 68) (> 21.90)	P-value^2^
Energy, kcal	2925.89 $\pm$ 460.94	2884.18 $\pm$ 868.69	2839.63 $\pm$ 470.71	0.44
Protein, % of energy	13.31 $\pm$ 1.90	14.50 $\pm$ 1.66	15.09 $\pm$ 2.05	< 0.001
Carbohydrate, % of energy	59.16 $\pm$ 5.30	57.51 $\pm$ 4.73	58.21 $\pm$ 5.43	0.19
Fat, % of energy	28.66 $\pm$ 5.15	29.52 $\pm$ 4.63	28.37 $\pm$ 5.72	0.39
Cholesterol, mg	259.60 $\pm$ 11.98	287.21 $\pm$ 11.78	299.07 $\pm$ 11.89	0.061
SFA, gr	26.96 $\pm$ 0.71	28.37 $\pm$ 0.70	26.72 $\pm$ 0.71	0.20
MUFA, gr	25.91 $\pm$ 0.84	28.23 $\pm$ 0.82	28.48 $\pm$ 0.83	0.06
PUFA, gr	30.38 $\pm$ 0.97	28.65 $\pm$ 0.95	26.45 $\pm$ 0.96	0.02
Vitamin C, mg	91.26 $\pm$ 6.05	137.01 $\pm$ 5.95	172.01 $\pm$ 6.01	< 0.001
Vitamin A, RAE	7432.57 $\pm$ 67.72	1058.32 $\pm$ 66.62	1515.03 $\pm$ 67.25	< 0.001
Thiamin, mg	2.68 $\pm$ 0.04	2.63 $\pm$ 0.04	2.63 $\pm$ 0.04	0.56
Riboflavin, mg	2.07 $\pm$ 0.07	2.31 $\pm$ 0.06	2.53 $\pm$ 0.07	< 0.001
Niacin, mg	28.67 $\pm$ 0.41	27.71 $\pm$ 0.41	26.34 $\pm$ 0.41	< 0.001
Vitamin B6, mg	1.36 $\pm$ 0.05	1.70 $\pm$ 0.05	1.80 $\pm$ 0.05	< 0.001
Vitamin E, mg	34.60 $\pm$ 1.36	30.32 $\pm$ 1.34	26.21 $\pm$ 1.35	< 0.001
Folate, mcg	237.50 $\pm$ 9.79	320.22 $\pm$ 9.63	390.99 $\pm$ 9.72	< 0.001
Vitamin B12, mcg	3.81 $\pm$ 0.17	4.46 $\pm$ 0.17	5.03 $\pm$ 0.17	< 0.001
Magnesium, mg	245.52 $\pm$ 6.67	239.02 $\pm$ 6.56	325.39 $\pm$ 6.62	< 0.001
Calcium, mg	1199.56 $\pm$ 39.88	1316.06 $\pm$ 39.23	1494.61 $\pm$ 39.60	< 0.001
Total fiber, gr	15.19 $\pm$ 0.44	19.74 $\pm$ 0.43	23.34 $\pm$ 0.44	< 0.001
Vitamin K, mcg	111.16 $\pm$ 6.98	153.46 $\pm$ 6.87	199.34 $\pm$ 6.93	< 0.001

1 Values are Mean ± SE. Energy intake and macronutrients were adjusted for age and sex; all other values were adjusted for age, sex and energy intake. Abbreviations: DPI: dietary phytochemical index; SFA, Saturated fatty acids; MUFA, Monounsaturated fatty acids; PUFA, Polyunsaturated fatty acids.

2 P-value obtained from ANCOVA test for adjustment of energy intake.

**Figure 1:**
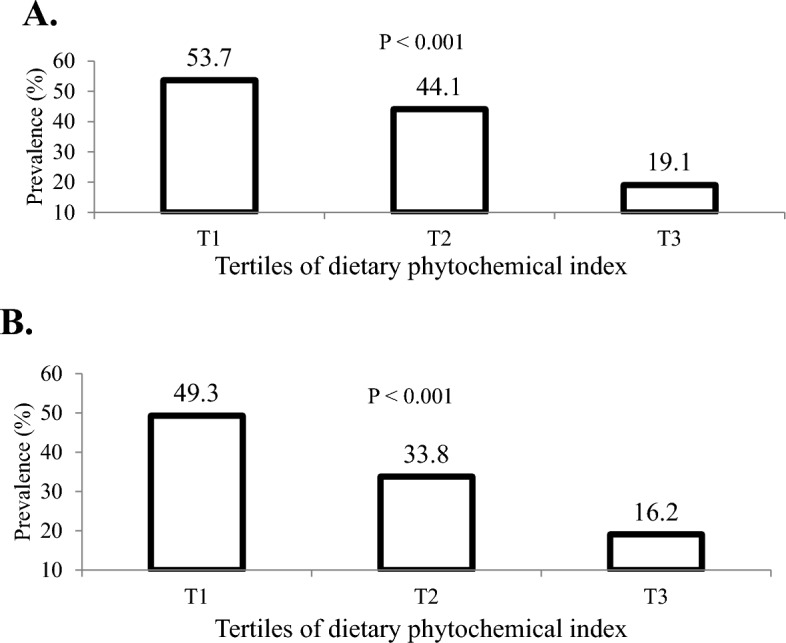
Prevalence of MUO across tertiles of DPI: (**A**) based on IDF definition, (**B**) based on IDF/HOMA-IR definition.

Figure 1 represents the distribution of adolescents with MUO phenotype across DPI tertiles. According to IDF definition, 53.7%, 44.1%, and 19.1% of adolescents were recognized with MUO phenotype in tertiles 1, 2, and 3 of DPI, respectively (P < 0.001). While, based on IDF/HOMA-IR definition, 49.3%, 33.8%, and 16.2% of adolescents were known as MUO in tertiles 1, 2, and 3 of DPI, respectively (P < 0.001).

Multivariable adjusted odds ratio (OR) and 95% confidence interval (95% CI) of MUO across DPI tertiles are summarized in Table 3. Based on both definitions of MUO, participants in the third DPI tertile had 80% lower odds of MUO, compared to those in the first tertile of the crude model (OR = 0.20, 95%CI 0.09–0.44). After adjustment for confounding variables, the association attenuated, but remained significant. In the fully-adjusted model, 61% (OR = 0.39, 95%CI 0.16–0.91) and 67% (OR = 0.33, 95%CI 0.13–0.83) reduced odds of MUO were observed in adolescents in the highest DPI tertile, compared to those in the reference tertile, based on IDF and IDF/HOMA-IR criteria, respectively. Furthermore, a significant inverse association was found per one tertile increase in DPI and odds of MUO based on IDF (OR = 0.35, 95%CI 0.43–0.98) and IDF/HOMA-IR (OR = 0.41, 95%CI 0.37–0.92) definitions.

**Table 3:** Multivariable adjusted odds ratio (OR) and 95% confidence interval (CI) for MUO across energy-adjusted tertiles of DPI^1^.

		DPI tertiles								
		T1 (n = 67) (< 11.50)		T2 (n = 68) (11.50–21.90)		T3 (n = 68) (> 21.90)		P-trend		Per one tertile increase
MUO phenotype based on IDF criteria										
Cases (n)	36		30		13					
Crude	1		0.68 (0.34–1.34)		0.20 (0.09–0.44)		< 0.001		0.47 (0.32–0.67)	
Model 1^2^	1		0.81 (0.40–1.65)		0.23 (0.10–0.52)		< 0.001		0.50 (0.34–0.74)	
Model 2^3^	1		1.02 (0.48–2.14)		0.38 (0.16–0.88)		0.03		0.64 (0.42–0.97)	
Model 3^4^	1		0.99 (0.47–2.10)		0.39 (0.16–0.91)		0.04		0.65 (0.43–0.98)	
MUO phenotype based on IDF/HOMA-IR criteria										
Cases (n)	33		23		11					
Crude	1		0.53 (0.26–1.05)		0.20 (0.09–0.44)		< 0.001		0.45 (0.31–0.67)	
Model 1^2^	1		0.60 (0.29–1.25)		0.20 (0.09–0.49)		< 0.001		0.47 (0.31–0.71)	
Model 2^3^	1		0.73 (0.34–1.56)		0.32 (0.13–0.79)		0.02		0.58 (0.37–0.90)	
Model 3^4^	1		0.70 (0.32–1.51)		0.33 (0.13–0.83)		0.02		0.59 (0.37–0.92)	

^1^All values are odds ratios and 95% confidence intervals.

^2^Model 1: Adjusted for age, sex, and total energy intake.

^3^Model 2: Additionally, adjusted for physical activity and socioeconomic status (parental education, parental job, number of family members, having car in the family, having computer/laptop, having personal room and having trip).

^4^Model 3: Additionally adjusted for body mass index (BMI).

Table 4 shows sex-stratified multivariable-adjusted odds ratios and the 95% CIs of being MUO across DPI tertiles. In the crude model, girls in the highest DPI tertile, in comparison to those in the bottom tertile, had 83% (OR = 0.17, 95%CI 0.05–0.54) and 88% (OR = 0.33, 95%CI 0.03–0.45) reduced odds of MUO based on IDF and IDF/HOMA-IR criteria, respectively. In the fully-adjusted model, adolescent girls in the highest DPI tertile showed a significant 75% (OR = 0.25, 95%CI 0.06–0.98) and a marginally significant 77% (OR = 0.23, 95%CI 0.05–1.09) lower risk of MUO according to IDF and IDF/HOMA-IR definitions, respectively. In boys, a significant inverse relationship between DPI and MUO was found in the crude model, based on IDF (OR = 0.23, 95%CI 0.08–0.67) and IDF/HOMA-IR definitions (OR = 0.27, 95%CI 0.09–0.77); however, these associations became insignificant after adjustment for all potential confounders.

**Table 4:** Multivariable adjusted odds ratio (OR) and 95% confidence interval (CI) for MUO across energy-adjusted tertiles of DPI, stratified by sex^1^.

DPI tertiles					
	T1 (< 11.50)	T2 (11.50–21.90)	T3 (> 21.90)		P-trend
MUO phenotype based on IDF criteria					
Girls					
(Cases/participants)	20/37	17/35	5/30		
Crude	1	0.80 (0.32–2.02)	0.17 (0.05–0.54)		0.01
Model 1^2^	1	0.79 (0.29–2.15)	0.16 (0.05–0.57)		0.01
Model 2^3^	1	0.84 (0.30–2.36)	0.25 (0.06–0.97)		0.07
Model 3^4^	1	0.84 (0.30–2.36)	0.25 (0.06–0.98)		0.07
Boys					
(Cases/participants)	16/30	13/33	8/38		
Crude	1	0.57 (0.21- 1.55)	0.23 (0.08–0.67)		0.01
Model 1	1	0.59 (0.19–1.82)	0.21 (0.06–0.72)		0.01
Model 2	1	1.23 (0.35–4.34)	0.42 (0.11–1.62)		0.18
Model 3	1	1.09 (0.30–3.93)	0.35 (0.09–1.40)		0.12
MUO phenotype based on IDF/HOMA-IR criteria					
Girls					
(Cases/participants)	18/37	11/35	3/30		
Crude	1	0.48 (0.19–1.27)	0.12 (0.03–0.45)		0.01
Model 1	1	0.43 (0.15–1.25)	0.11 (0.03–0.49)		0.01
Model 2	1	0.49 (0.16–1.46)	0.21 (0.04–0.98)		0.04
Model 3	1	0.49 (0.16–1.47)	0.23 (0.05–1.09)		0.05
Boys					
(Cases/participants)	15/30	12/33	8/38		
Crude	1	0.57 (0.21–1.57)	0.27 (0.09–0.77)		0.01
Model 1	1	0.67 (0.22–2.05)	0.28 (0.08–0.93)		0.04
Model 2	1	1.41 (0.39–5.02)	0.43 (0.58–0.15)		0.38
Model 3	1	1.28 (0.35–4.66)	0.49 (0.12–1.99)		0.29

^1^All values are odds ratios and 95% confidence intervals.

^2^Model 1: Adjusted for age, and total energy intake.

^3^Model 2: Additionally adjusted for physical activity and socioeconomic status (parental education, parental job, number of family members, having car in the family, having computer/laptop, having personal room and having trip).

^4^Model 3: Additionally adjusted for body mass index (BMI).

## Disscussion

Findings of this cross-sectional study suggested that according to both definitions of IDF and IDF/HOMA-IR, the prevalence of MUO among Iranian overweight and obese adolescents was more than 30%. Higher dietary intake of phytochemicals was significantly related to a reduced risk of being MUO, according to both definitions. In addition, the association was more powerful for adolescent girls than boys. This was the first study examined the association of DPI, as a representative index of the phytochemical content of diet, and metabolic health status of adolescents.

Adolescence is an important life stage in determining later metabolic health status and associated complications. Some evidence shows that cardiometabolic profile of adolescents with MHO phenotype persists into adulthood; suggesting that they probably have a lower chance of developing cardiovascular diseases in their later life stages^13^. Due to the failure of various weight loss strategies in youth, it can be more advantageous to obtain a favorable metabolic health status in adolescence and maintain it until adulthood. Some findings have shown that lifestyle contributors, such as physical inactivity and unhealthy dietary behaviors, might result in a metabolic unhealthy profile in obese or overweight individuals^37,38^. According to our study results, increasing dietary intakes of phytochemical-rich foods including fruits, vegetables, legumes, nuts, and whole grains could be a beneficial approach to achieve and possibly maintain MHO phenotype in overweight or obese adolescents.

Despite the importance of phytochemical intake in health and disease, limited investigations have assessed the association of dietary phytochemicals consumption and metabolic health among adolescents. In contrast to our findings, intake of polyphenols was not substantially related to MetS or its components among European adolescents, although it was inversely associated with BMI^24^. However, the mentioned study has only examined dietary polyphenols in relation to odds of MetS and total DPI has not been considered. Some other investigations have suggested that higher dietary phytochemical consumption during adolescence could be related to lower risk of cardiometabolic factors, later in life. Two previous cohort studies indicated that more flavonoid intake from vegetables and fruits during adolescence is related to elevated insulin sensitivity (HOMA2-%S) in adulthood and decreased LDL-c concentrations only in women^23,39^. Though, further prospective investigations are worthwhile to explore the association between dietary phytochemical intake from various sources during teenage years and future metabolic health status. Furthermore, a number of previous studies have investigated dietary phytochemical intake in relation to MetS among adults and obtained contradictory results. A recent cross-sectional study in Yazd, a province in center of Iran, found a negative relation between higher adherence to a phytochemical-rich diet and odds of MetS, especially among females^40^. A similar report has been released from a study performed among the Polish adult population, indicating that higher dietary polyphenol intake was inversely associated with odds of elevated blood pressure and diabetes in females^41^. However, DPI was not significantly related to chance of MetS in another study among Iranian adults^42^. Possibly, differences in study population, the exposure of interest, study design, and criteria used to define metabolic health status can explain the inconsistencies of these results.

The negative relation between DPI and metabolic health of adolescents can be explained by several mechanisms. Some studies demonstrated that oxidative stress and inflammation could have important roles in pathogenesis of unhealthy metabolic status^43–45^. Accumulating evidence exists on the anti-inflammatory properties of phytochemical compounds. For instance, it has been demonstrated that phytochemicals such as resveratrol, quercetin, genistein, kaempferol, and daidzein could inhibit pro-inflammatory responses in macrophages by downregulating interleukin-6, interleukin-1β, tumor necrosis factor-α, and could inhibit nitric oxide by regulating inflammatory cascades mainly NF-κB and mitogen-activated protein kinase (MAPK) signaling pathways^46–49^. In addition, anti-inflammatory cytokines such as transforming growth factor-β1 or interleukin-10 could be induced by phytochemicals^50,51^. Also, many phytochemicals have been found to have antioxidant properties via directly scavenging reactive oxygen or reactive nitrogen spices (ROS/RNS), chelating metal ions, or enhancing expression of antioxidant enzymes including catalase, glutathione peroxidase and superoxide dismutase^52^.

Several limitations of our study should be acknowledged for interpreting the findings. First, this study had a cross-sectional nature that could not represent the causal link between DPI and MUO in adolescents. Reverse causation could be another possible concern in such cross-sectional studies, since individuals with cardio-metabolic disorders symptoms might change their dietary intakes. To establish the casual relationship between DPI and MUO, prospective cohort studies are required. Second, although a number of confounders such as physical activity, sex, age, BMI, energy intake, and socio-economic status have been controlled in this study, uncontrolled confounders such as sleep dissatisfaction or insufficient sleep, psychological and environmental factors might affect our results. Third, DPI has been computed according to the calories of the consumed foods; thus, some phytochemical-rich foods without calories, such as tea, have been neglected. Fourth, no previous study has been validated DPI in comparison to phytochemical biomarkers. However, a number of previous studies documented the associations between this score and various diseases^53–56^. Such associations between this index and diseases can be considered as an equivalent strategy to validate the score. Fifth, evaluation of dietary intakes was subject to recall and misclassification bias, despite using a valid and reliable questionnaire. Our study had several strengths, as well. We evaluated PDI in relation to metabolic health status of adolescents for the first time. In addition, outcome assessment was performed by objective methods. Nevertheless, we should cautiously extrapolate our study findings to other populations, as dietary intakes could vastly vary among nations. Therefore, well-designed prospective studies are needed among different populations to shed a light on the relation between phytochemical content of diet and metabolic status of adolescents.

In summary, we found a negative association between higher intake of phytochemical-rich foods and chance of MUO in overweight and obese Iranian adolescents, especially girls. To confirm our findings, further prospective investigations are required.

## Data Availability

The datasets used and analysed during the current study available from the corresponding author on reasonable request.

## Abbreviations

DPI: Dietary phytochemical index
FFQ: Food frequency questionnaire
BMI: Body mass index
IDF: International Diabetes Federation
IR: Insulin resistance
MHO: Metabolically healthy obese/overweight
HOMA-IR: Homeostasis Model Assessment Insulin Resistance
MUO: Metabolically unhealthy obese/overweight
WHO: World Health Organization
PAQ-A: Physical Activity Questionnaire for Adolescents
SES: Socioeconomic status
WC: Waist circumference
DBP: Diastolic blood pressure
SBP: Systolic blood pressure
FBG: Fasting blood glucose
TG: Triglycerides
HDL-c: High density lipoprotein cholesterol
SFA: Saturated fatty acids
ANOVA: Analysis of variance
ANCOVA: Analysis of covariance
SE: Standard error
SD: Standard deviation
95% CI: 95% Confidence interval
OR: Odds ratio
